# An integrative review on children's perceived and experienced subjective digital well-being

**DOI:** 10.3389/fdgth.2025.1410609

**Published:** 2025-03-14

**Authors:** Halla Björk Holmarsdottir, Idunn Seland, Liudmila Zinoveva, Monica Barbovschi, Alina Bărbuță, Dimitris Parsanoglou, Maria Symeonaki

**Affiliations:** ^1^ Department of Primary and Secondary Teacher Education, Oslo Metropolitan University, Oslo, Norway; ^2^ School of Governance, Law and Society, Tallinn University, Tallinn, Estonia; ^3^ Institute of Sociology, Romanian Academy, Cluj-Napoca, Romania; ^4^ Department of Social Work, Babeș-Bolyai University, Cluj-Napoca, Romania; ^5^ Department of Sociology, National and Kapodistrian University of Athens, Athens, Greece; ^6^ Department of Social Policy, Panteion University of Social and Political Sciences, Athens, Greece

**Keywords:** digital activities, online activities, children's perspectives, digital well-being, subjective well-being

## Abstract

This review examines children's perceived and experienced subjective digital well-being by investigating their digital activities, behaviours and online relationships across three domains (Family, Leisure, and Education) presenting children's own perspectives. The included studies are limited to research published between 2011 and 2021 using European samples incorporating children aged 5–17 years. While research on children's digital well-being has expanded over the last two decades, the novelty of this review is that it presents research across all activity domains, representing an ecological approach to child development, one that aims to capture children's own views. The 23 studies identified for the review show, first, an apparent shortage of studies on children's well-being involving digital technologies that incorporate children's own perspectives on their situation. Second, the review shows that these studies relate primarily to well-being outcomes categorised as either social, emotional and cultural outcomes or as cognitive development and educational outcomes. Directions for further research on children's digital well-being are suggested.

## Introduction

Growing up in the 21st century means children are immersed in a digital culture that presents such new opportunities as connecting with peers or accessing educational resources instantly. However, legitimate concerns exist about the challenges faced by the omnipresence of digital technology in the everyday lives of children, as well as its effects on their overall subjective well-being. A key characteristic of “21st-century children” (1) is their “hyper-connectedness” (2) from an early age and the potential risks this poses for well-being, which is crucial during our formative years, shaping children's futures, including their health, work and social lives (3). Yet, we still lack definitive evidence of whether digital technology poses a risk to children's overall subjective well-being and mental health in general (4).

The predominant displacement hypothesis (5) suggests that the negative effects of digital activities are due to the replacement of alternate activities, such as socialising with peers and family, reading books or exercising. In their research, Przybylski and Weinstein (6) test an alternate theory, the digital Goldilocks hypothesis, which they suggest moderates the use of digital technology to be non-intrinsically harmful and instead to have a potentially positive effect on children's mental well-being. Thus, the digital Goldilocks hypothesis identifies the moderate or “just right” use of technology as advantageous in an increasingly digital world, and it appears supported by a systematic review of the literature (7), suggesting the most robust studies actually show a U-shaped relationship between time spent using digital technology and the impact on children's mental well-being. This U-shaped relationship means that no use and excessive use can both have small negative impacts on mental well-being, while moderate use can have a positive impact (7).

How to develop, nurture and sustain well-being and inclusion as lifelong processes and to enable all children to find positive pathways to adulthood (8) is thus a crucial question. Well-being over the life course is shaped by innate and situational indicators (developmental, psychosocial, digital and physical environment) and individual lifestyle choices; accordingly, maintaining well-being requires a comprehensive life course approach to prevention across various fields, as well as a focus on individual and social ecosystems surrounding the everyday lives of children. In this review, we follow the United Nations (9), Article (1) definition of children as individuals “below the age of eighteen years”, and so our review includes children aged 17 years and under. As an integrative review, we focus our attention to children's own views on their digital well-being.

### A framework for measuring well-being

The concepts of “health” and “well-being” are intrinsically linked and often used interchangeably. However, only health is authoritatively defined, while well-being is used and understood in different ways and contexts (10). More specifically, research applying measures of well-being may be viewed as incorporating various dimensions, drawing on insights from different academic disciplines, including psychology, medical sciences, economics and sociology (3). However, the different dimensions of well-being among children should not be treated as separate and should instead be viewed as developing alongside each other (3). Thus, understanding these dimensions and how they develop requires an approach that looks at several microsystems in children's everyday lives that affect their psychological, cognitive and social development (11, 12). For instance, the school can be an important microsystem for identification and prevention and for disclosing cognitive development and educational outcomes, but it can also help uncover social and emotional outcomes linked to such factors as bullying, in which the teacher plays a crucial role (13). Other important microsystems are the family and the wider community, including children and young people's leisure activities. Thus, measuring and defining well-being calls for an approach that considers children's lives in the present and that is connected to the ecosystems surrounding them (3, 12).

Moreover, well-being has historically been viewed at a societal level with a focus on healthy, productive societies (14) measured through proxies of well-being such as income, literacy and life expectancy as well as subjective measures on how life is perceived and experienced by individuals (15, 16). One approach to measuring perceptions and experiences has been through the focus on *subjective well-being* (SWB), which is defined as “a person feeling and thinking his or her life is desirable regardless of how others see it” (17). An example following this definition regarding digital technology and life satisfaction (or lack thereof), subjective well-being could be experienced by a teenager being active on social media and assessing her number of followers. In addition, this definition highlights the *thinking* and *feeling* dimensions of SWB:

• *Feeling* refers to the emotional/affective dimension of SWB, with a prevalence of positive emotion over negative emotion leading to higher SWB.
• *Thinking* refers to the evaluative/cognitive dimension of SWB, where the evaluation of individuals' lives in predominantly positive terms leads to higher SWB (18).

According to Twigg et al. (19), psychological well-being is an increasingly important issue in research on children's lives and, more specifically, on the distinction between hedonic and eudaimonic well-being. Twigg et al. (19) point out:

[H]edonic well-being is defined as the subjective experience of pleasure or happiness, involving both an affective component (i.e., positive and negative emotions) and a cognitive dimension relating to elements of (and overall) quality of life… [while] eudaimonic definitions are focused on notions relating to self-purpose, self-fulfilment, sense of autonomy and good relations with others.

Diener (17) points out that eudaimonic well-being does not reflect the individual's subjective judgment but the value framework of the researcher. In this review, we chose not to focus on eudaimonic well-being but rather on hedonic well-being, allowing us to focus on children's feelings and thinking as expressions about their subjective well-being. Focusing on the hedonic dimension of well-being means that researchers can include children's own lived experiences, allowing them to become active participants in research that affect them directly instead of asking others about them, such as parents or teachers. This leads to the following review question for the current study: How does the literature present children's perceived and experienced subjective well-being in using digital media within and across the contexts of family, school and leisure time?

### Data and methods

The present study builds on a re-examination of the literature identified for a scoping review conducted as part of the (DigiGen – The impact of technological transformations on the Digital Generation) (2020–2022), funded by the European Union Horizon 2020 programme (20). The original scoping review aimed to analyse why some children and young people seem to reap the benefits of digitalisation while others seem to be impacted negatively. Central to this analysis were the restraints caused by digital divides (21–25), individual and structural inequalities (26) and risk and vulnerability (27–30) to enable research on children's agency, their social relationships and their meaning-making involving digital technology across the different domains of their everyday lives. The idea of re-examining the literature for children's own perspectives on their well-being in using digital media then emerged and motivated the current integrative review.

The integrative review does not merely report on previous literature but critically analyses and synthesises research in the current field such that new perspectives on the topic are generated (31). In mature research fields, the integrative review may help bridge fragmented ideas not sufficiently informing and relating to each other, proposing new research foci (32). In the current review, children's well-being, an established outcome variable in research on children and young people's use of digital technology, is studied from a novel angle that points to a new research frontier involving the viewpoints of the research objects. The integrative review may thus be part of an iterative cycle, where a body of primary research provides the descriptive basis but is then critically assessed for where the evidence is scarce to guide new primary research and theory development (31).

### Information sources

Table 1 displays the three separate literature searches (Family; Leisure; Education) that were conducted using six databases, as follows:

• EBSCO (Academic Search Ultimate, Education Source, SocIndex, ERIC)
• Web of Science Core Collection
• Applied Social Sciences Index & Abstracts (ASSIA) (ProQuest)
• PsychInfo
• Social Care Online
• Science Direct

**Table 1:** Overview of databases searched per context, with dates for most recent search.

Database	Context	Date for most recent search
EBSCO: (Academic Search Ultimate, Education Source, SocIndex, ERIC)	Family; Leisure; Education. Added from EBSCO for Education: Teachers' Reference Center	Family: 5 May 2021
		Leisure: 27 Sep. 2021
		Education: 8 Sep. 2021
Web of Science Core Collection	Family; Leisure; Education	Family: 3 May 2021
		Leisure: 28 Sep. 2021
		Education: 9 Sep. 2021
Applied Social Sciences Index & Abstracts (ASSIA) (ProQuest)	Family; Leisure; Education	Family: 5 May 2021
		Leisure: 29 Sep. 2021
		Education: 9 Sep. 2021
PsychInfo	Family	4 May 2021
Social Care Online	Family	19 May 2021
Science Direct	Education	8 Sep. 2021

### Eligibility criteria

Following the prerequisites of the (DigiGen – The impact of technological transformations on the Digital Generation), this review maintains a European focus linking policy to research on children's digital well-being by including only research based on data including European children aged 5–17 years. Previous research has revealed a lack of studies on the use of digital technology among the youngest children (33). The Better Internet for Kids (BIK)^1^ initiative and the European Strategy for a Better Internet for Kids (BIK+)^2^ both emphasise digital literacy and online safety, shaping national policies in this area reinforced by the General Data Protection Regulation (GDPR)^3^ and the EU's Digital Services Act,^4^ as they include provisions specifically addressing children's online experiences. This body of policy frameworks is expected to influence empirical research on children's digital well-being, delimiting our sample to Europe while at the same time maintaining this review's policy relevance. Our timeframe 2011–2021 captures key shifts in children's digital engagement, from early EU studies (EU Kids Online)^5^ to the rise of social media and updated policies like Better Internet for Kids before major legislative changes in 2022. Table 2 displays the inclusion criteria for the original review (34).

**Table 2:** Inclusion criteria for current scoping review.

Inclusion criteria
Studies must cover children’s digital activities, behaviours and relationships online
Time span publication: 2011–2021 (no data from before 2006)
Geographical area: Europe
Studies must have been published in peer-reviewed journals
Studies must be in English

### Search

Table 3 displays the search strings for all contexts (Family; Leisure; Education) used for the four EBSCO databases (Academic Search Ultimate, Education Source, SocIndex, ERIC).

**Table 3:** Search strings applied to the EBSCO databases.

Search	Family	Leisure	Education
Title	[ICT* or digital* or online* or internet* or (screen) W1 time or (social or new) W1 media or sharent*] AND (home* or parent* or famil*)	[ICT* or digital* or online* or internet* or screen W1 time or (social or new) media or device*] AND (entertain* or communic* or negotiat* or connect* or play* or digital W1 space or socialization* or creat* or collaborat* or content W1 shar*) AND (child* or kid* or young* or youth* or adolesc* or teen*)	(ICT* or digital* or internet* or online* or computer*) AND (teach* or learn* or classroom* or (primary or secondary) W1 (education or school) or (elementary or middle) W1 (education or school)
Abstract	(child* or kid* or young* or youth* or adolesc* or teen*) AND (age* or gender* or boy* or girl* or sociodem* or socioec* or migrant* or immigrant* or ethnic* or minority* or unemploy* or (high or low) W1 income or inequal* or single W1 parent or co-parent* or cultur* or risk* or vulnerab* or marginalise* or disab* or disadvant* or special W1 (needs or education) or LGBT* or (rainbow or patchwork) W1 family or foster W1 parent or homeless* or heterosex* or homosex* or urban* or rural*)	(age* or gender* or boy* or girl* or sociodem* or socioec* or migrant* or immigrant* or ethnic* or minority* or unemploy* or (high or low) W1 income or inequal* or single W1 parent or cultur* or risk* or vulnerab* or marginalise* or disab* or disadvant* or special W1 (needs or education) or LGBT* or heterosex* or homosex* or urban* or rural*)	(child* or young* or youth*or adolesc* or teen* or pupil* or student*) AND (competenc* or skill* or literacy* or transition* or instruction* or pedagog* or didact* or activ* or interactive* or homework* or collaborative* or class* or practice* or hybrid* or (distance or remote) W1 learning or achievement* or BYOD* or (formal or informal) W1 learning) AND (age* or gender* or boy* or girl* or sociodem* or socioec* or migrant* or immigrant* or ethnic* or minority* or unemploy* or (high or low) W1 income or inequal* or single W1 parent or cultur* or risk* or vulnerab* or marginalise* or disab* or disadvant* or special W1 (needs or education) or LGBT* or heterosex* or homosex* or urban* or rural*)
Title or abstract	–	(PISA or EU kids online)	(ICILS)

Table 3 shows that the search strings for the contexts Family, Leisure and Education were applied differently to the search in Title and Abstract in the databases. This strategy was the result of an initial trial-and-error approach to see what combination of keywords yielded the most fruitful results. A complementary search was run in the EBSCO databases for Education, combining the complete search string for both Title and Abstract with “ICILS” (aimed at studies reporting on the International Computer and Information Literacy Study) and for Leisure with “PISA or EU kids online”. The abbreviation BYOD in Table 3/Education stands for “bring your own device”.

### Selection of evidence sources

The results from all database searches were imported first into EndNote, organised in three separate libraries for Family, Education and Leisure and then imported from EndNote to the online screening tool Rayyan (https://www.rayyan.ai), where duplicates were removed. Rayyan allows for two (or more) researchers to read and assess titles and abstracts in parallel blind mode before switching off blind mode and comparing results. This was done by hand by three separate teams consisting of two researchers, with one researcher (second author) being the same for all teams to maintain coherence and an overview of the screening process. At this stage, the screening teams assessed the title and abstract, applying the inclusion criteria (see Table 2) when these were present in the title or abstract, or both, and without discriminating between data sources or evidence types. The screening teams met in pairs at the beginning, mid-way and at the end of the screening process to compare and discuss results until agreement was reached.

### Data charting process

After the screening in Rayyan was completed, the teams were expanded with more researchers to chart the preliminarily included studies and assess these using the inclusion criteria (see Table 2). One Excel worksheet was set up for each charting team and made available in Google Docs, and the preliminarily included studies were divided among the researchers. Each researcher retrieved and then read the assigned studies to fill out the following characteristics sufficiently in the Excel sheet:

• Author
• Year
• Title
• Journal
• Country (data sample)
• Research question
• Population (age group)
• Sample size
• Methodology
• Source of data (evidence)
• Duration
• Digital device investigated in study
• Representations of digital divides
• Representations of well-being outcomes
• Key findings

### Critical appraisal of individual evidence sources and continuation for current review

The charting teams, spending three months on this process, met at the beginning, mid-way and at the end of the period to discuss their assessment until an agreement was reached.

For the current review, the body of studies resulting from this data charting process were re-examined to determine if (1) the study's research questions sought to understand children's views, opinions or experiences in relation to their own use of digital technologies, (2) if the study presented children's own views, and (3) how the study presented children's views to support the study's theoretical interpretation of data. The sample of studies identified from this re-examination were then assessed to see how the data presented *children's hedonic well-being* (19) meaning their pleasure or happiness—or its opposites—involving both an affective component and a cognitive dimension. We were paying particular attention to whether the studies provided negative cases, i.e., narratives that did not fit the studies' identified themes or theoretical framework. Additionally, we assessed how well the analysis in the identified studies explained why the children felt in the described ways. Finally, we categorised the identified studies according to data collection methods.

## Results

**Figure 1:**
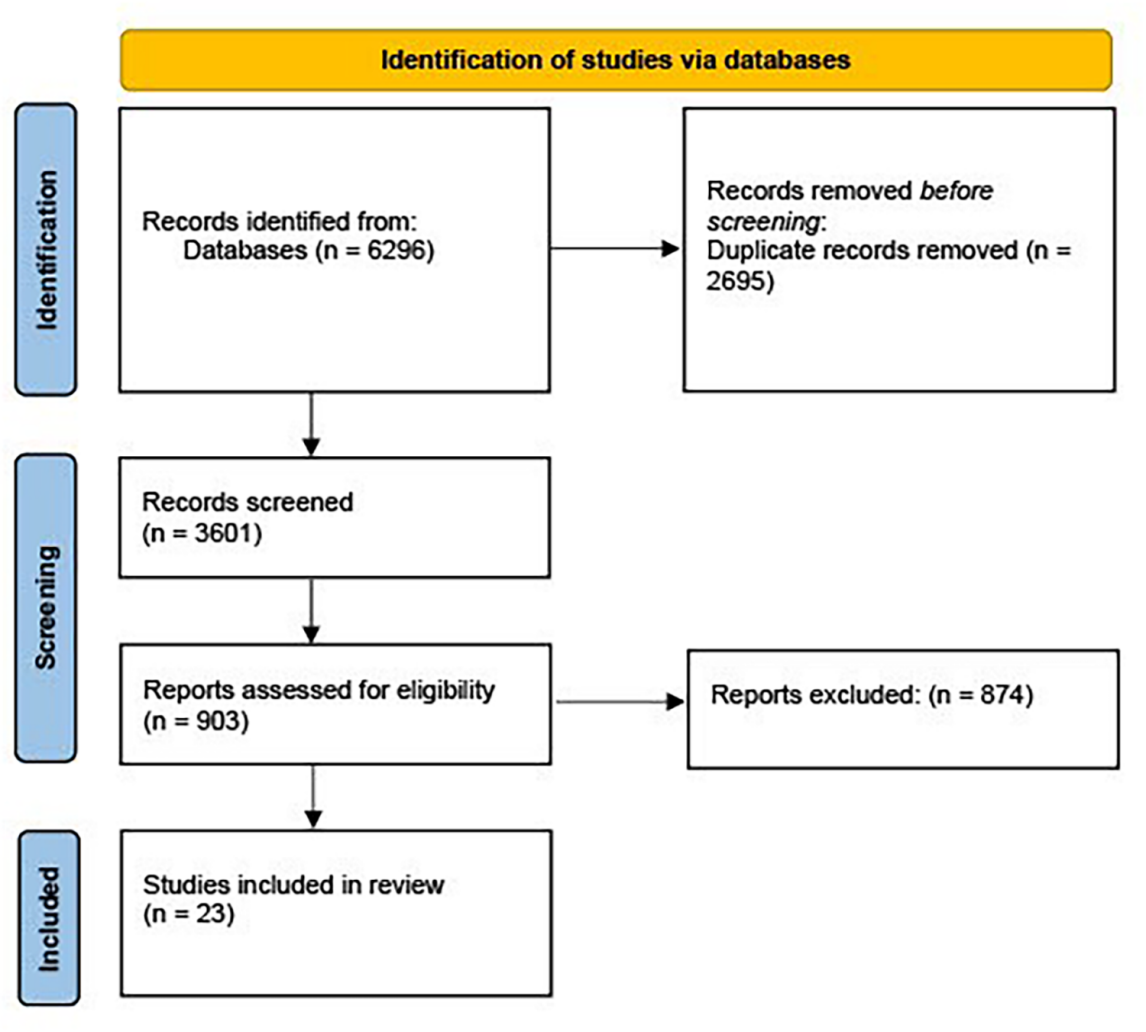
Flow diagram for scoping review including searches of databases. Derived from: Page et al. (68).

The numbers of sources of evidence screened, assessed for eligibility, and included in the review, are displayed in Figure 1.

### Data overview

Focusing on studies with a particular emphasis on children's digital activities within the family in European countries, Table 4 shows the characteristics for seven included articles published between 2013 and 2021.

**Table 4:** Selection of sources for family activities and relationships.

Year	Author	Country	Method	Age group (years)	Sample (N)
2013	Navarro et al. (36)	Spain	Survey	10–12	1,068
2017	Brito & Diaz (35)	Portugal	Interview	3–8	25
2018	Trumello et al. (38)	Italy	Survey	10–21	743
2021	Miltuze et al. (40)	Latvia	Survey	8–11	261
2020	Sarkadi et al. (37)	Sweden	Survey	4–15	68
2020	Twigg et al. (19)	United Kingdom	Survey	10–15	7,596
2021	Romera et al. (39)	Spain	Survey	10–13	866

Table 5 shows the characteristics for seven included articles on leisure activities and relationships, published between 2012 and 2021.

**Table 5:** Selection of sources for leisure activities and relationships.

Year	Author	Country	Method	Age group (years)	Sample (N)
2012	Aarsand (41)	Norway	Interview	16–17	32
2012	Marsh (42)	United Kingdom	Children’s panels, interviews, diaries, video recordings	5–11	36
2018	Nasaescu et al. (44)	Spain	Survey	11–19	2,139
2018	Wernholm (47)	Sweden	Interview	8–12	9
2019	Gomez-Baya et al. (43)	Spain	Survey	13–16	882
2020	Savci et al. (46)	Republic of Türkiye	Survey	14–18	549
2021	Eek-Karlsson (45)	Sweden	Interview	14–15	32

Table 6 displays the characteristics of the nine included studies on learning activities, attitudes and relationships published between 2011 and 2021.

**Table 6:** Selection of sources for learning activities, attitudes and behaviours.

Year	Author	Country	Method	Age group (years)	Sample (N)
2011	Mylläri et al. (54)	Finland	Interview	11–18	27
2014	Liljeström et al. (50)	Sweden	Digital storytelling	6–12	32
2016	Mota et al. (48)	Portugal	Survey	13–16	64
2017	Vasalou et al. (49)	United Kingdom	Video recordings	11–12	8
2015	Bjørgen & Elstad (56)	Norway	Video recordings, interview	9–13	37
2017	Camilleri & Camilleri (51)	Malta	Interview	Secondary school	41
2018	Kajamaa et al. (53)	Finland	Video recordings	9–12	94
2020	Cranmer (52)	United Kingdom	Interview	13–17	7
2021	Buško & Bezinovic (55)	Croatia	Survey	15–19	4,492

Overall, these results show that remarkably few studies include self-reported or subjective child data, with 23 studies in total on European samples included from 2011–2021. We have incorporated surveys and questionnaire studies that could be argued as including self-reported data, although the young respondents are only able to pick one pre-defined answer to represent their view. Within this limited number of studies, even smaller number refers to children's actual statements, retrieved either from interviews, diaries or from video observations.

## Results of individual evidence sources

### Family activities and relationships

Digital activities within the family context present a dynamic landscape shaped by a combination of school policies, parental perceptions, and the children's own preferences for entertainment and learning (35). In a study by Brito and Dias, some children exhibit autonomy in using digital technologies for homework, highlighting a growing independence in incorporating technology into their learning processes at home. A 6-year-old child described her way for retrieving images from the Internet:

Child: I wrote ‘lion’ and then I went to the image, and it showed up on the screen. I pressed the left button of the mouse, and then it showed up ‘copy image’.

Focusing on diverse Internet activities and communication purposes, Navarro et al. (36) investigate the impact of Internet usage and parental mediation on online victimisation among Spanish children attending rural public schools. Participants in the study, aged 10–12 years, were queried about their engagement in Internet-related activities. The findings indicate that nearly the entire sample (*n* = 1,068) utilised the Internet weekly, primarily for information-seeking purposes. This was followed by children's activities, such as chatting, emailing and visiting their profiles on social networks. In addition, more than half of the children reported engaging in activities, such as downloading music and videos, instant messaging, and online gaming. As demonstrated in the research, parents may not be effectively enforcing age restrictions on social networks, as children reported accessing these platforms despite be prohibited for minors under 16 years old. Alongside such factors as time spent online and Internet communication, the authors emphasise the significance of understanding online communication motivated by social compensation and the desire to make new friends, as these factors contribute to heightened risks of online victimisation (36). In fact, children who reported being victims of cyberbullying indicated three reasons for talking online: “daring to say more”, “belonging to a group or being a member of something” and “making new friends”, while non-victims reported that “speaking with real-life friends” was a significantly more common reason for engaging in online conversations.

Digital activities in families can also include not only what children and young people do themselves but also what other members of the family do that can affect them, such as when family members share photos or experiences from joint family activities online, often referred to as “sharenting”. The study by Sarkadi et al. (37) aims to understand what children and young people think about sharenting via a survey of 68 children in Sweden aged 4–15 years. “Sharenting” was described as either “send a picture to a relative”, “take a photo without permission”, “post pictures of children on social media” and “write things about children on social media”. The children overall reported having largely negative attitudes towards sharenting, and they expressed wanting to be asked and listened to before parents share stories or images of them on social media. These young children reported that they also want to be asked before photos are taken of them in general, perhaps realising a sign that they recognise their rights in this regard. One interesting aspect of the study is that children aged 4–6 thought it *least acceptable* that a photo of them was taken without their permission, suggesting that while many parents might think this is an acceptable practice, even for children as young as these, it is unacceptable. For children and young people, having clear boundaries around sharenting and their privacy is important. Thus, the children convey a noticeably clear message: “wanting to be asked and listened to before their parents “sharent”, that is share stories or images of them on social media” (37).

Relationships are an essential part of identity development for children and young people. Moreover, digital technology can be a crucial element in the development of their personal identity. While peers are important, children’s relationships with their parents can also play an important role in identity development. Trumello et al. (38) investigate the relationship between children and young people and their parents by focusing specifically on emotion regulation and callous-unemotional traits related to Internet addiction. Their study is based on self-report measures of relationships with parents (both mothers and fathers) and emotional regulation (in its two dimensions: cognitive reappraisal and expressive suppression), callous-unemotional traits (in its three dimensions: callousness, uncaring, and unemotional) and Internet addiction among 743 Italian adolescents aged 10–21 years (M_age_ = 15.64). In their study, children who score higher on the Inventory of Callous–Unemotional Traits (ICU) in the self-report questionnaire (traits linked to lack of empathy, guilt, and emotional expression) show a highly positive association with Internet addiction. In addition, the study includes self-reported data from children and young people using two versions of the Lum Emotional Availability of Parents (LEAP) questionnaire to illustrate the connection between Internet addiction and the emotional quality of the relationships children and young people have with their parents. The results show that children and young people with lower scores on perceived emotional availability with their mothers as opposed to their fathers are associated with higher levels of Internet addiction (38). This suggests that availability and perhaps even quality in parent–child relationships are crucial for children’s self-regulatory use of digital technology, in addition to the need for the development of sufficient emotional and self-regulatory processes.

Romera et al. (39) delve into the dynamics of specific online activities that may play a mediating role in the relationship between child disclosure vis-à-vis their parents of the children’s online activities and their role in cyberaggression. By posting messages about others online, children normalise hurtful behaviour as a means to uphold their social position and status within the peer group. Romera et al. (39) emphasise the importance of addressing this type of online behaviour because, although it is not initially intended to cause harm, it is susceptible to misunderstandings and, therefore, increases the risk of engagement in cyberaggressive behaviour. The results of the survey, which utilises self-reporting and involves 866 primary school children in Spain, show that children feel understood and supported by their parents in a climate of trust and communication within the family environment, pointing to the importance of parent–child relationships. This encourages open communication about Internet use, which, in turn, helps reduce involvement in risky online behaviour (39).

Another longitudinal study by Miltuze et al. (40) investigates Internet use among primary school-aged children, exploring general parenting practices (such as emotional warmth and inconsistent parenting), parenting strategies related to Internet use (establishing rules, exerting control, forbidding behaviours) and the child's perception of the quality of the child–parent relationship, all in association with the child’s ratings of compulsive Internet use (CIU). The study shows that child-reported positive relationships with parents serve as crucial protective factors against problematic Internet use. Moreover, while a favourable relationship with parents deters problematic Internet use, clear and consistent parental rules function as preventive measures. However, parental forbidding and technical controls may inadvertently increase CIU, emphasising the need for balanced approaches to digital parenting strategies (40).

The data in the longitudinal study by Twigg et al. (19) include children aged 10–15 years in sampled households in the United Kingdom who were invited to complete a youth self-completion questionnaire, while household members aged over 16 completed their own detailed interview, either face-to-face with an interviewer or through a self-completion online survey. Measuring children's social media usage included self-report responses to time spent chatting or interacting with friends online through social media during a normal school day. The results of this self-reported data show that for children, their life satisfaction decreased over time as children got older, and a detrimental effect is found for girls more so than boys and for children whose mothers experience poor mental health. Children who reported high social media use, more than 7 h per day, also show lower life satisfaction than children who reported no social media use, while moderate social media use (i.e., anything less than 3 h per day) was unrelated to changes in life satisfaction scores over time. Children were also asked how well they felt supported by their family, whether they talked with their parents about things that mattered to them and whether their parents were interested in how they did at school. Based on their results from the self-reported data, Twigg et al. (19) conclude that a supportive family environment may offer some protection against worsening life satisfaction scores and that a mother's mental health status was detrimental to changes in life satisfaction scores over time.

### Children’s perspectives: family activities and relationships

Our review includes studies in which children describe, both affectively and cognitively, how they can incorporate digital technology into their daily lives. For instance, the study by Brito and Dias (35) includes reflections from a 6-year-old child describing a sense of mastery and making the technology “come to life”. While the study points to co-presence activities and activities conducted under parental supervision, it also shows children exhibiting a certain degree of autonomy and, in some respect, a blurring of child–parent roles (35). Several studies in our review focus on family relationships and activities, pointing to the importance of healthy and supportive relationships as a critical factor in children's overall well-being in relation to digital activities (19, 38–40). For some children, being online provides them with support and a sense of belonging or allows them to keep in touch with one's friends (36). What is less in focus, however, is how parents’ use of online platforms that may actually harm children's privacy. Sarkadi et al. (37) include the perspectives of children aged 4–6, who argue it is unacceptable for a photo to be taken of them without their permission, suggesting that while many parents might think this an acceptable practice, for very young children, it is not.

### Leisure activities and relationships

One of the main areas of ICT use is leisure activities, including gaming, as well as communication with peers and friends and content consumption on social media and digital platforms. In the field of gaming, research with teenagers in Norway (41) has highlighted the ways in which children and young people reflect on their online leisure activities and position themselves as players. Based on eight focus-group interviews with 32 teenagers, the paper examines how teenagers take stances on digital games and how they deal with discourses of concern when presenting their own playing. Another strong point of the article is its focus on how “adult” stereotypes are used in teenagers’ discussions of playing digital games. In the—most often externally imposed—dichotomy between “hardcore” and “casual” players, a third figure, that of a “knowledgeable” player, appears in the discourse of adolescents who seem to understand but also act upon existing risks within gaming. At another level, teenagers tend to define and present themselves as ordinary players, i.e., as an alternative to the hardcore and the casual player: The implication of viewing ordinariness in such broad terms is that deviant and troublesome positions, such as the hardcore player, are less likely to be found among teenagers. Rather, what are seen among teenagers are variations on ordinariness. Ordinariness, however, is not constructed in a social and cultural vacuum, it is related and adjusted to discourses of concern, but on *teenagers' own terms* (41): 974, emphasis added).

For example, one teenage girl positions herself as “in the know” regarding playing games while avoiding the controversial stance of being a “hardcore” player. By mentioning that she does not play online with unknown co-players and that her game console is old and not connected to the Internet, she creates an image of her gaming habits as “unproblematic”, non-threatening and controlled: “…I can sit by myself for hours and just play those old games…” (41). Another boy positions himself as well-aware of his choice of games. To him, playing is unproblematic, but it may pose a problem for “someone”. Some teenagers display themselves as experts, while simultaneously claiming to be ordinary players. For instance, one boy positions himself as reflective and conscious when he acknowledges “actually (…) we maybe have been playing a little too much”.

In a paper discussing the research conducted in two primary schools in England, children were not only perceived as active agents, but also as a significant factor in the research process. As Marsh (42), puts it, “It was important that children's involvement in the project was active, as their experiential knowledge of their own practices was crucial in addressing the research questions”. Using the concept of “knowledge brokering” as an analytic device, the nature of children's contribution to participatory research studies as mediators of their own culture was examined. One interesting observation from the study is that the children themselves certainly felt they were more appropriate researchers of children's cultural (including media and playgrounds) practices than adults. When asked why children's involvement in data collection was important, participants emphasised that their peers would feel more at ease talking to them, as one girl reflected:

Child: Talk to our friends and…when you’re friends with someone, it’s like they tell you more stuff; they’re not, like, bothered if they say something wrong. But if someone else, like an adult, talks to them, they’re like….

Another boy suggested that children might gather more naturalistic data compared to adults:

Child: Yeah, I think it does because, like, you know, like, if you were filming other children, they’ll try really hard to really impress you. If it’s another child, they’ll play it like they’ll normally play and not try and add anything extra special in.

Apart from the methodological insights the article offers, examples of children's creativity and capability to combine traditional playground games and rhymes with their media culture are highlighted.

Communication and socialisation among children and young people have become effective to a great extent in online spaces. Everyday communication, peer relations, friendship maintenance and social support, but also stigmatisation and victimisation occur throughout online interactions. A longitudinal study, in which 882 adolescents (aged 13–16 years) from Andalusia (Spain) participated, shows that communication with friends is more frequent online than offline (43). Moreover, more frequent text messaging is related to greater ease in making friends and avoiding bullying in adolescents with more initial difficulties. The article concludes that “the use of online communication with friends suggests more benefits than risks” (43).

Similar results are obtained by another cross-sectional study with a representative sample of 2,139 adolescents enrolled in 22 secondary schools in Andalusia, which addresses the issue of abuse of technology among adolescents (44). The study shows that high levels of social and emotional competences are related to less technology abuse. Moreover, using emotional content in online communication is related to a greater abuse of technology, without excluding contradictory outcomes:

On the one hand, this might suggest that adolescents with good social and emotional competencies, who decide to express these competencies online have an increased need to use and possibly abuse technology. On the other hand, it is possible that a high level of social and emotional competencies might be related to an excessive expression of emotions online and these highly emotional interactions might also be related to the abuse of technology [(44)].

Digital spaces constitute in general an arena where children and young people can construct and perform their social identity. Popularity, for example, is accrued in digital spaces through “tagging”, which is translated physically to a higher status in school. Eek-Karlsson (45), in a study of 32 boys and girls aged 14–15 years from two schools in Sweden, suggests that children negotiate their social identity depending on what is regarded as “normative” in the specific context. By their acts, they construct frames for what is considered appropriate for boys/girls and, simultaneously, they perform their social identity. Without denying differences between online and offline communication, the article analyses normative expectations in relation to different areas and processes, such as group membership, gender performances and the intertwining among gender, sexuality and group status. According to the study, both boys and girls learn how to define normality and regulate their identities through online interactions. As the author outlines, hidden discourses and positioning processes become visible online, making it an effective tool for both preserving and challenging normality. Children learn to balance seeking positive attention and avoiding various forms of exclusion. On the one hand, as one boy states, “Social media is mostly about the outside…your outward face in society, not much about the inside at all…about making a good image”. On the other, it is important to present oneself online in the same way as offline, such as by not publishing “strange” photos of oneself, as one girl explains: “It is as if someone would see me in reality…this is the kind of photos we publish”, which also involves embracing vulnerability.

Another study that involved 549 adolescents (296 girls and 253 boys) investigates the relationship among family life satisfaction, problematic social media use (PSMU) and social connectedness (46). Using three relevant scales, i.e., the Social Media Disorder Scale, the Social Connectedness Scale and the Family Life Satisfaction Scale, each of which includes several items, the analysis demonstrates that problematic use of social media directly and indirectly negatively predicted social connectedness. Problematic social media use also directly and negatively predicted family life satisfaction. However, family life satisfaction predicted social connectedness, and problematic social media use predicted social connectedness via family life satisfaction.

…both problematic social media use and family life satisfaction should be taken into consideration in studies related to increasing social connectedness. PSMU may reduce family life satisfaction, and low family life satisfaction may result in lower levels of social connectedness (46).

Wernholm (47) explore how nine Swedish children (8–12 years) learn from informal digital participation during leisure, playing online games. Driven by friendship, interest, knowledge or performance, the children learn language, concepts, artefacts, produced digital content and shared and distributed knowledge:

Child: … when you play with someone older and really good at English and I ask him what the others are saying … and then you learn.

Child: You can imitate someone who does it a right way

Wernholm (47) concludes that children's informal participation in digital arenas should be understood as a social process of learning and that teachers may build on and connect these experiences to children's learning in school.

### Children’s perspectives: leisure activities and relationships

From the reviewed literature we see that for older children and adolescents using digital technology during leisure time, the “thinking” or evaluative/cognitive aspect of their experienced well-being gains importance while there is still play and creativity. Thus, being “knowledgeable” or even an expert in gaming means mastery of certain skills (41), and the children in Wernholm (47) talk of learning from playing games in a broad sense. The children participating in the study by Marsh (42) participate in what the author labels “knowledge brokering”, where children are essential mediators of their own culture, and the adolescents talking to Eek-Karlsson (45) are knowledgeable about how they perform their identity on social media, and especially what they should not do when going online. Interestingly, the positive affective aspect of using social media for making friends (Gomez-Baya et al. (43) is balanced by the negative affective aspect of technology abuse (44, 46). Feeling bad when going online may cause teenagers to abuse digital technology.

### Education activities, attitudes and behaviours

Mota et al. (48) used an open-ended questionnaire to investigate how 64 students aged 13–16 years experienced using a software to explore statistical data (TinkerPlots) during three school lessons. The students, who were recruited from three schools in a socioeconomically disadvantaged area in Lisbon, Portugal, had no previous experience using digital technology in mathematics in the school context. Many of the 64 students reported that the software facilitated their statistical learning, exemplified as follows: “Yes, because I have never been able to understand boxplots and through this software, I was able to” and “I started to improve my learning with graphs.”. The students were divided into collaborative groups during the intervention. In total, 54 of the 64 students report enhanced learning due to help from peers: “We could think more and better organise with peers” and “[…] we were able to learn with our peers what we did not know”. Mota et al. (48) conclude that using the software in groups increases students’ perseverance behaviour during tasks, possibly adding to their mathematical resilience, which in turn helps their academic performance. However, a residual number of students reported feelings of distress or indifference after the three lessons, which meant their statistical literacy was not enhanced from use of the software. Students’ lack of successful learning is explained by a lack of technical skills (48).

Using video recordings over a period lasting three weeks, Vasalou et al. (49) investigate how a digital game (Words Matter) facilitates social interaction to foster and shape learning of word decoding, spelling and fluency among eight British 11–12-year-olds with dyslexia. The children naturally engaged in constant “game talk”, i.e., discussions related to game performance, content, actions and experiences, in contrast to previous research demonstrating that children with dyslexia often avoid disclosing their learning problems:

Student: I get the hardest words! Look how long my words are!

Student: My words would once cover up the whole page.

Vasalou et al. (49) also observe that children's free navigation of Words Matter strengthened their agency in the learning process, increasing their engagement in learning and the facilitation of peer tutoring:

Student: We need time to do it [voices out a game strategy].

Student: You have to do it quickly [voices out the correct game strategy].

The children's “game talk” served to normalise unsuccessful attempts at specific game tasks, while breakdowns in the game voiced through “game talk” prompted instructions from the teacher. However, whether learning breakthroughs emerged from these situations depended wholly on the quality of the instruction. In addition, Vasalou et al. (49) observe that engagement with the game caused children to concentrate more intensely on smaller and competitive elements instead of transferring their skills to new tasks in the game, which could have expanded their learning.

In a qualitative study of the impact of digital storytelling and the emerging learning ecosystem on learning processes (50), 32 students aged 6–12 years in Finland were invited to use digital cameras (their own or the school's) and were asked to make a video-based diary at the end of each school day. The qualitative analysis of the videos produced by students in terms of structure, commentary and descriptions revealed a high degree of agency and self-directedness among the students, even the youngest. Based on the findings, the authors argue that inquiry-driven learning tasks and afforded learning resources are valuable, enabling students to form their own complex study processes. Finally, the study argues in favour of recognising students’ agency in design-oriented pedagogy and co-opting children and young people in the co-development of learning processes and the creation of local knowledge through storytelling.

Camilleri and Camilleri (51) use semi-structured, face-to-face interviews with 41 secondary school students in Malta to investigate their discernment towards and preconceptions of the in-class use of digital games, stories and simulations, suggesting that students are increasingly acquiring skills and competences from blended learning. However, the study also revealed mixed results regarding the students' experiences, as some exhibited increased skills and competence and reported improved critical thinking, decision-making skills and teamwork, as the following excerpts show:

Student: I learned from the digital story task. It was a positive experience for me when I composed a digital story together with my classmates.

Student: This digital storytelling experience has provided me with the opportunity to reflect on my life experiences.

Yet, other students remained wary of the usefulness and ease of use of playing digital games at school. The authors reflect on possible differences in skills and motivation as a result of gender, age and socioeconomic status differences, and they encourage practitioners in education to consider integrating digital games into the courses' learning outcomes and curriculum programmes.

Within the context of inclusive education policies, an exploratory, participatory research study was designed to gain insights into how visually impaired children experience digital technologies in learning (52). Semi-structured interviews were conducted with seven children with aged 13–17 in three schools in England to gain accounts of their activities of and experiences with digital use practices. Using social practice theory, results were analysed to identify practices that children characterised as digital learning and digital accessibility. The children described some of the benefits of using digital technologies for their learning practices:

Student: It’s quicker to use, less of a hassle to carry, easy to enlarge things. It’s just generally better

The children also spoke of the challenges and constraints they face in their digital learning practices. The conclusions of the study are thus mixed; on the one hand, young people recognise the benefits of digital technologies for learning; on the other, the study cautions about the necessity of inclusive training for teachers to prevent the deployment of potentially stigmatising practices and an extra task load for the students to overcome barriers.

In a study of the mediating effect of digital learning environments on students' funds of knowledge and knowledge creation, the authors (53) analyse 111 h of video records of 9–12-year-old Finnish students' (*N* = 94) making and design activities, collected during one semester in a novel learning environment called the FUSE Studio. Often, students' knowledge creation focused on following the structures and instructions given by the FUSE computer program and the facilitating teachers. However, at times, students used their own initiative to break away from the situation creatively (referred to as “horizontal knowledge breaking”):

Student: Hey, this wasn’t good … This is really difficult.

Student: Try it!

Student: Oh, now I know! I’m going to make a contemporary one.

Student: So, what are you doing?

Student: I’m designing my kitchen sink.

Student: Show me what you’re doing.

Student: This is going to be a modern one. The faucet is going to be in the middle of the sink, it’ll be a circle, and water will come out of each side.

In some cases, the creative tensions between students' funds of knowledge and the rules and instructions of formal schooling led to an innovative process in which student groups and sometimes also students with their teachers collectively challenged and questioned the existing rules of knowledge and led them to co-configure more expanded, future-oriented knowledge (i.e., “knowledge expansion”). The implications of the study are in line with the direction of expanding learning-enhancing opportunities via digital platforms and solutions.

Myllari et al. (54) present a five-month intervention in four Finnish schools, where 27 students in total aged 11–18 years are taught to use concept-mapping software at appropriate stages of the teaching–studying process and across diverse subjects. The aim was to investigate aspects of student motivation and self-regulation for incorporating digital technology into the school context. As an illustration of student motivation, one girl reports after working with the software: “It’s nicer to look for information yourself. You might learn more that way’. As for self-regulation, one girl, checking information on Google, says: “…and if I find some nice article, I try to look for more information from that (…) and it gets more fun all the time [laughs]”. It should be noted that students were recruited for the study by their teachers, mostly based on students' ability to work independently.

In April 2020, during the extraordinary event of the COVID-19 school lockdowns, Buško et al. (55) used an online survey with Likert and frequency scales on a sample of 4,492 Croatian students aged 15–19 years to examine relevant sources of stress regarding online classes. While the results show variety in the stress experienced by respondents, having a heavy workload due to many assignments across subjects with strict deadlines, missing live lectures, learning difficult academic content and having uncertainty about exams are some of the salient sources of stress perceived, mainly among girls and students in the highest grades. Moreover, uncertainty about returning to school and missing being with friends add to the intensity of short term-emotional and psychosomatic difficulties experienced. Buško et al. (55) suggest that a lack of information from relevant policy makers may have signified a greater problem for students' well-being during school lockdowns than the actual changes in living and online learning conditions.

In a normalised school and home situation and by using qualitative interviews with and video observations of 37 Norwegian children aged 9–13 years, Bjørgen and Erstad (56) study how children understand and engage with digital practices between school and leisure. In technology-rich school contexts, children are introduced to formal digital competences, enhancing their digital literacies at both locations. Children thus display identities as agentic learners, using the digital competences acquired at school to continue learning at home. Citations from two children, a girl and a boy, illustrate how they were first introduced to software at school and then used the programme at home of their own volition to make covers for schoolbooks or write their own books on themes of personal interest, such as pets.

Student: […] it is for instance to learn to use new things that might be useful at school and for homework and stuff, as for instance Its Learning, Power Point. Many students don’t know how to use this yet..

Student: I make covers for schoolbooks and the like, or for school projects and the like, or just drawings on Paint if I don’t have anything else to do.

Interviewer: What do you find the most exciting?

Student: Covers, because there are so many strange images that can be used (…) And it is possible to paste in images, for example cars or the like (…) I can change colour and such.

The study reveals that by providing opportunities for engagement in explorative, competence-productive practices nurturing identity and agency, schools can enhance learning by utilising digital tools that blend into children's informal and familiar practices.

### Children's perspectives: education activities, attitudes and behaviours

The results show that when exposed to digital technology in learning situations, children's subjective well-being centres on cognitive responses for learning and affective responses both from experiencing mastery in using technology to solve novel tasks, and cut-off from learning due to insufficient digital competence or lack of interventions from teachers in critical situations. These are highly contrasting reports of subjective well-being. Spontaneous feelings of creativity brought on by digital technology in diverse learning situations can bring children joy (49, 53), support self-directed learning and spur motivation to find information and learn from it (54, 56). However, not all interventions trying out digital technology in the classroom were successful, with outcomes such as distress, indifference (48) or hesitant about the usefulness of such activities (51). The literature thus shows that while using digital technology for learning can benefit some, it can harm learning for others.

The observational study by Vasalou et al. (49) provides a nuanced understanding of learning and social interaction, wherein dyslexic children engage deeply in a game meant to enhance their understanding of words. However, they also help each other through complex parts of the game, thus facilitating peer learning, something also uncovered by Mota et al. (48). However, the study also clearly shows that if the teacher does not provide the correct scaffolding during the learning process of the game, the child will not learn from breakdowns; instead, the child will want to continue the game (49). The importance of teachers' competence is also pointed out by Cranmer (52).

## Discussion

Our review aimed to uncover children's thinking and feelings about their subjective well-being as it relates to their digital everyday lives across three domains, namely family, leisure and education. More specifically we were concerned with understanding how the literature presents children's perceived and experienced well-being in using digital media within and across these domains. The limited literature incorporating data on children's perspectives of how their digital activities, behaviours and relationships affect their subjective well-being falls under two areas (1) Social, emotional and cultural well-being and (2) Cognitive and educational well-being. These two areas illustrate the feeling and thinking dimensions of subjective well-being as proposed by Diener (17, 18), where feeling refers to the emotional/affective dimension and thinking refers to the evaluative/cognitive dimension. Thus, these two dimensions are well suited to studying subjective well-being within these specific empirical contexts (family, leisure and education). Studies identified for this review demonstrate that children's experiences using digital technology often bridge all these activities, i.e., between the family environment and the learning environment, both in school and during leisure time.

### Social, emotional and cultural well-being

Ivari (57) reminds us that “…existing research has argued children are the experts in “being kids”, and this expertise needs to be available for the development of digital technology aimed at them”. Building on children's sense of mastery may go a long way towards aiding in the development of exciting and valuable digital solutions for children. While the idea of children being digitally competent and knowing more than adults has been challenged, we also see a polarised response to the adoption of digital technology by children, especially young children. This polarised debate sees parents and others (e.g., teachers and policymakers) act with extreme concern or with overly optimistic enthusiasm.

Yet, concerns are real, and children must develop competences to protect themselves. This requires we also address children's thinking and feelings about their subjective well-being as it relates to their use and experiences with digital technology. Our review has shown that even victimised children can find support online, and staying connected with friends can be an essential part of growing up in a rapidly changing digital world. Conversely, children are not the only ones who may need to develop digital competences, as many parents are not fully confident in their own digital skills (58). One area of concern in which even incredibly young children have expressed knowledge and concern with adult behaviour is parental *sharenting* (59), which not only holds negative connotations but also disregards children's rights (60). Sharenting can also affect children's feelings regarding their well-being as this is something they may also have little control over. Still, for many parents, “digital technology represents the single most noticeable difference between their own childhood and that of their children” (58). What is clear from our review and the literature in general is that digital technology is associated with agency, both for children and parents, but the importance of family values and bringing up children in ways that link these values will continue to be significant (58, 61).

Generally, leisure activities linked with digital devices are often debated in terms of screentime and concerns with content and contact risks. Gaming and screentime debates are thus intricately linked. Our review has shown that some children described feelings of risks to well-being by spending too much time gaming, while others feel that gaming is an essential part of establishing their identity and as such promotes a sense of well-being (41). While our review pointed to the importance of gaming and girls' identity development, a great deal of research has been conducted on the relationship between boys and technology, particularly in terms of the identity that arises from these relationships and implications for learning (62, 63). What is clear from our review and other studies as well is that research on girls as gamers is scarce (64), which may be explained partly by the fact that males have traditionally designed video games and been stereotyped as “gamers” and even as “hardcore” players, an identity with which girls are reluctant to identify (41). This reluctance to identify as a “hardcore” player can demonstrate how girls evaluate their subjective well-being in predominantly positive terms, which could lead to higher subjective well-being (17). The ambiguity of social media and the difficulty of partly preserving one's façade in one's surroundings and partly normalising an imperfect life can be described as a type of *networked privacy*, which “invokes the constellation of audience dynamics, social norms and technical functionality that affect the processes of information disclosure, concealment, obscurity and interpretation within a networked public” (65). According to Marwick and boyd (65), this requires an understanding of the context (the leisure context through social media) in which the information is shared, but it also shapes the context, which, according to Marwick and boyd (65), is regularly blurred and can collapse. However, others point out that these contexts are also porous. As such, children's interactions within them construct mesosystemic interactions that include a range of participants that requires skills in dealing with networked privacy (66).

### Cognitive development and educational well-being

Digital technology has been at the forefront of education as far back as the popularisation of the radio in the 1920s, with increases in the use of digital technology taking place over roughly the last 40 years. A significant focus has also been on improving learning and providing support to those with special needs. What our review shows is that the use of software for learning and even educational games has been beneficial to some while simultaneously harming learning for others (48–50). Notably, this pertains not only to children with learning problems or disabilities. On the one hand, young people recognise the benefits of digital technologies for learning, but on the other, the literature cautions about the necessity of inclusive training for teachers to prevent the deployment of potentially stigmatising practices and extra task loads to overcome barriers when it comes to children with special needs (52). Other studies in our review provide examples of how creativity within the school context, made possible by the use of digital tools for learning (horizontal vs. “traditional” vertical knowledge expansion) and how digital tools may facilitate peer collaboration and peer learning (53). Creativity and playing with digital technology may enhance competence, and the technology enables children to pursue other interests, such as drawing, creating or investigating while using the technology as a tool (54, 56). For children, the ability to creatively play around with technology can demonstrate the thinking aspect of subjective well-being and relate to a kind of “life evaluation” in which they can evaluate their life experiences (e.g., life satisfaction) as something positive (17). In addition, studies of gaming have uncovered the social processes of learning, for example, learning English from those who are better at it (47). Recent EU projects, such as DigiGen [see (67)], have pointed to some of the same results, especially those referring to learning English through gaming, using digital technology to enhance competency and contributing to self-directed learning.

## Conclusion and suggestions for further research

This article shows an apparent shortage of studies on children's well-being involving digital technology that incorporate children's own perspectives on their situation. For a notable share of the 23 studies identified for inclusion in the review, children's views were collected through structured questionnaires, limiting children's freedom to express their perspectives by choosing between pre-defined options set by adult researchers. Future research on children's well-being should aim to consider children's own views. Recent research shows that children want to be asked what they think and adults to truly hear their responses (67).

For the qualitative studies using interviews diaries and observations, the child perspectives relate to hedonistic well-being (19), underlining fun, a sense of ease, creativity and sociability connected with the use of digital tools. However, the included literature also reveals that children aged below 18 years are experiencing obstacles, difficulties and disappointments online. While representing the counterpart to hedonistic well-being, *overcoming* such obstacles and disappointments could lead to a state of eudaimonic well-being (19). Additionally, children's digital activities often have a purposeful element, such as making friends and staying connected, which closely links to life satisfaction according to other models and theories (see for example (67). This purposeful behaviour suggests that children's engagement with digital technology is not only for hedonistic pleasure but also serves meaningful purposes in their lives.

The studies included in this review do not sufficiently address the purposeful aspects of children's digital activities or how overcoming online challenges can contribute to eudaimonic well-being. Although two studies within learning activities point to how teachers bear the main responsibility for ensuring that digital technology is used in ways that enable learning for *all* students, a more in-depth discussion is warranted. Future research should explore the purposeful behaviours and the potential for digital activities to contribute to both hedonistic and eudaimonic well-being, taking into account children's own perspectives and experiences.

## Footnotes

^1^Better Internet for Kids: https://better-internet-for-kids.europa.eu/en (accessed on 2 February 2025).

^2^A European strategy for a better internet for kids (BIK+): https://digital-strategy.ec.europa.eu/en/policies/strategy-better-internet-kids (accessed on 2 February 2025).

^3^General Data Protection Regulation (GDPR): https://gdpr-info.eu/ (accessed on 2 February 2025).

^4^The EU’s Digital Services Act: https://commission.europa.eu/strategy-and-policy/priorities-2019-2024/europe-fit-digital-age/digital-services-act_en (accessed on 2 February 2025).

^5^EU Kids Online: https://www.lse.ac.uk/media-and-communications/research/research-projects/eu-kids-online (accessed 2 February 2025).
